# Rosmarinic acid ameliorates the complications of monocrotaline-induced right ventricular hypertrophy on the left ventricle: Investigating the signaling pathway of Wnt/β-catenin in the heart

**DOI:** 10.22038/IJBMS.2024.75201.16301

**Published:** 2024

**Authors:** Narges Atefipour, Mahin Dianat, Mohammad Badavi, Maryam Radan, Seyyed Ali Mard

**Affiliations:** 1 Department of Physiology, Faculty of Medicine, Ahvaz Jundishapur University of Medical Sciences, Ahvaz, Iran; 2 Persian Gulf Physiology Research Center, Basic Sciences Research Institute, Ahvaz Jundishapur University of Medical Sciences, Ahvaz, Iran

**Keywords:** β-catenin, Monocrotaline, Right ventricular – hypertrophy, Rosmarinic acid, Vascular calcification, Wnt

## Abstract

**Objective(s):**

Right ventricular hypertrophy (RVH) often results in failure of the right ventricle or even the left ventricle. Rosmarinic acid (RA), a natural polyphenol, is commonly found in Boraginaceae species and some species of ferns and hornworts. This study looked at how RA affects oxidative stress and left ventricular hemodynamic functions as well as RVH in monocrotaline (MCT) induced RVH model rats.

**Materials and Methods:**

To cause RVH, MCT (60 mg/kg) was intraperitoneally (IP) injected. Rats were given saline or RA (10, 15, and 30 mg/kg, gavage, over 21 days). In anesthetized rats, the lead II electrocardiogram was recorded. The hemodynamic functions of the isolated heart were measured using the Langendorff apparatus (at constant pressure). Investigations were made into the right ventricular hypertrophy index (RVHI), the activities of superoxide dismutase, catalase, glutathione, and Wnt and β-catenin gene expressions in the left ventricle. H&E staining was used.

**Results:**

A significant decline in electrocardiogram parameters and anti-oxidant enzyme activities, an increase in QTc (Q-T corrected) intervals, MDA (Malondialdehyde), RVHI, and Wnt/β-catenin gene expression, and also significant changes in the hemodynamic parameters were demonstrated in the MCT group. RA improved the above-mentioned factors.

**Conclusion:**

According to the findings, RA may act as a cardioprotective agent against cardiovascular complications brought on by RVH due to its capacity to boost the activity of cardiac anti-oxidant enzymes and decrease the expression of genes involved in vascular calcification.

## Introduction

Numerous pieces of evidence suggest that RVH is associated with a variety of diseases, including pulmonary hypertension, left ventricular pathology, and arrhythmogenic RV cardiomyopathy, leading to heart failure and ultimately death (1, 2). Although relatively limited information is available regarding RV function, the impairment of RV in various disease states and its impact on the outcome of these diseases suggests that RV function is important. Therefore, a deeper understanding of these issues is critical (3).

To develop new, efficient therapeutic strategies, it is still essential to understand the pathobiology of RVH. To this end, suitable animal models of RVH are required. The RVH rat model induced by monocrotaline (MCT) has helped researchers to better understand the development process of RVH (4).

According to earlier research, oxidative stress is a factor in the pathogenesis and development of RVH (5). Oxidative stress conditions increase the production of reactive oxygen species (ROS) as well as disrupt the normal functioning of the anti-oxidant system, ultimately causing mitochondrial dysfunction and cell death (6, 7). Plenty of studies indicate that living organisms contain anti-oxidant mechanisms, including catalase (CAT), glutathione (GSH), and superoxide dismutase (SOD), to protect cells against reactive oxygen species (ROS) (8). 

Vascular calcification is a threatening complication of cardiovascular diseases that affects tissues including arteries, heart valves, and heart muscle (9). The process known as vascular calcification (VC) involves the deposition of calcium phosphate crystals in the form of hydroxyapatite in the media and intima of the vascular wall. Vascular calcification reduces the elasticity of the arterial wall, changes the hemodynamic characteristics, and increases the risk of cardiovascular events (10). VC is associated with oxidative stress, including ROS (11). Vascular smooth muscle cells (VSMCs) differentiate under the influence of ROS. VSMCs undergo phenotypic changes in a peroxidized environment that cause vascular dysfunction, like vascular inflammation and calcification (12). Although Wnt/ β-catenin signaling has a crucial role in bone formation, there is also convincing evidence that it plays a significant role in VC (13).

Rosmarinic acid (RA) is a natural polyphenol and a caffeic ester found in some plants, including Rosmarinus Officinalis. The presence of phenolic-OH groups plays an important role in its anti-oxidant activity (14). Numerous studies have documented RA’s potent capacity to scavenge free radicals, and as a result, it is used to eliminate extra free radicals from the body (15). In addition, RA has numerous biological activities such as anti-inflammatory (16), cardioprotective and antidysrhythmic (17), hepatoprotective (18), antihypertensive (19), anti-depressant (20), and anti-oxidant effects (21).

When oxidative stress brought on by ROS leads to damage, the body’s endogenous anti-oxidants might not always be enough to protect it. As a result, in recent years, there has been a growing interest in the therapeutic potential of natural products as anti-oxidants in minimizing tissue damage brought on by free radicals (22, 23). 

Considering the effect of MCT on oxidative stress and incidence of RVH, since the left ventricle plays an important role in systemic blood circulation, this study aimed to investigate the anti-oxidant properties of RA and the complications induced by the RVH model on the left ventricle.

## Materials and Methods

***Materials***

 Xylazine (2%) and ketamine HCl (10%) were purchased from Alfasan Co. (Netherlands). MCT and RA were obtained from Sigma Aldrich (Germany). Catalase (CAT), superoxide dismutase (SOD), malondialdehyde (MDA), and glutathione (GSH) were bought from KIA ZIST Co. (Iran). Moreover, potassium chloride, sodium chloride, potassium hydrogen phosphate, sodium hydrogen carbonate, magnesium sulfate, calcium chloride, and D-glucose were purchased from Merck Co. (Germany). RNA extraction kit, cDNA synthesis kit; the housekeeping and target primers; and SYBR Green Master Mix were respectively purchased from Anacell Co. (Iran), Sinaclon Co. (Iran), and Ampliqon Co. (Denmark).

***Animals***

 Forty-eight Sprague-Dawley Male rats (180–200 g) were obtained from the animal center of Ahvaz Jundishapur University of Medical Sciences and were maintained under standardized conditions (12-hr light/dark cycle) with appropriate temperature and humidity and were allowed free access to food and water. The experiments were carried out following ethical guidelines. The protocol was confirmed by the local research committee at Ahvaz Jundishapur University of Medical Sciences (grant No. APRC-0013).

***Induction of right ventricular hypertrophy model***

 The right ventricular hypertrophy model was established in animals after 21 days of MCT (60 mg/kg, IP) injection to the target groups (24).

***Animals groups***

 For the purpose of this experiment, the animals were randomly divided into six groups of eight:

1) Control: saline (21 days, gavage) 

2) R30: receiving RA (30 mg/kg, 21 days, gavage)

3) MCT: receiving MCT (60 mg/kg, first day, IP) (24)

4) R10+ MCT: receiving MCT (60 mg/kg, first day, IP) (24)+ RA (10 mg/kg, from the first day for 21 days, gavage) (25, 26)

5) R15+ MCT: receiving MCT (60 mg/kg, first day, IP) (24)+ RA (15 mg/kg, from the first day for 21 days, gavage) (25, 26)

6) R30+ MCT: receiving MCT (60 mg/kg, first day, IP) (24)+ RA (30 mg/kg, from the first day for 21 days, gavage) (25, 26)

***Anesthesia induction***

 At the end of the testing period, anesthesia was induced using xylazine (10 mg/kg) and ketamine (50 mg/kg).

***Electrocardiography (ECG) recording method***

 In all groups, 15 min after anesthesia (to stabilize hemodynamic factors), ECG was recorded (lead II) using BioAmp and Power lab system (AD Instruments, Australia), electrocardiogram parameters including Q-T interval, QRS complexes, and HR (Heart Rate) were calculated (27). Since the Q-T interval is affected by the heart rate, Bazzet’s formula is used to calculate corrected Q-T (QTc) as follows:

Bazzet´s formula: Q-T corrected (QTc) ₌ Q-T interval /square root RR

***Heart isolation method***

 At the beginning of the experiment, to prevent coagulation, heparin (IP, 1000 units per kg of animal weight) was injected. Then the rats were ventilated by putting a cannula in the trachea and connecting it to a ventilator for rodents (UGO BASILE, model: 7025). After that, the abdominal cavity was opened, the diaphragm torn, the chest opened, an incision made in the aorta, and a steel cannula was inserted and secured with a suture. The heart was then removed and put into a Langendorff device (based on constant pressure) containing Krebs-Henseleit bicarbonate buffer (KCl 4.6 mM, NaCl 115 mM, KH_2_PO_4 _1.2 mM, NaHCO_3 _25 mM, MgSO_4 _1.2 mM, CaCl_2_ 2.5 mM, and glucose 11 mM in distilled water). Krebs perfusion buffer at pH=7.40 contained carbogen gas (95% O_2_ + 5% CO_2_), ata temperature of 37 °C. A power lab system (AD-Instruments, Castle Hill, Australia) monitored and recorded cardiac function (28).

***Hemodynamic functions in isolated hearts***

A latex balloon filled with distilled water was passed through the left atrium into the left ventricle and connected to a pressure transducer. Its performance was evaluated after 15 min. The end-diastolic pressure was then modified with a balloon volume change to produce an end-diastolic pressure of 5 mm Hg, and the Powerlab system analyzed the signal from the pressure transducer. We measured the following parameters (29):

Left ventricular end-diastolic pressure (LVEDP), left ventricular systolic pressure (LVSP), left ventricular developed pressure (LVDP= LVSP – LVEDP), RPP (product of LVDP and heart rate) as the indices of contraction and relaxation, maximum rate of fall (-dp/dt), maximum rate of rise (+dp/dt), and coronary flow were measured with the Power lab system.

***Assessment of anti-oxidant markers (GSH, CAT, SOD) and MDA***

Heart tissue (left ventricle) was examined to determine the activities of CAT, GSH, SOD, and MDA. The heart tissue was removed after the hemodynamic functions were assessed, placed in liquid nitrogen, and kept at -80 °C until the experiment. The frozen tissues were carefully weighed, homogenized at a ratio of 1:10 in phosphate-buffered saline, and centrifuged at 12,000 rpm for 15 min at 4 °C. The supernatant was taken out to measure the desired biochemical indices (27). The activity of CAT, GSH, SOD, and MDA was measured with diagnostic kits. 

***Evaluation of right heart hypertrophy***

For this purpose, after hemodynamic measurements were completed, the chest cavity was immediately opened and the heart was rapidly dissected and placed in ice-cold normal saline to remove blood. Then the atria were removed from the harvested hearts and the right ventricle was separated from the left ventricle + septum. Then, hypertrophy was evaluated using the Fulton index. The ratio of the weight (g) of the right ventricle divided by the weight (g) of the left ventricle + septum was calculated as a measure of RVH (Fulton index) (30).

***Histopathology of heart tissue***

Histopathological examination was carried out as described in previous studies (31). Briefly, rat hearts (whole hearts) from each group were fixed in 10% buffered formalin for an overnight period, paraffinized, cut into 4 μm sections, stained with H&E, and examined under a light microscope to detect pathological changes in the heart tissues.

***RNA extraction and cDNA synthesis***

RNA extraction from tissue samples (left ventricle) was done using a commercial RNA extraction kit. 

The purity and concentration of extracted RNAs were spectrophotometrically determined using a NanoDrop (Eppendorf, BioPhotometer Plus, Germany). Then, 1 μg of total RNA was used to synthesize cDNA with a commercial Kit. 

***RT-qPCR***

 Step-one systems (Applied Biosystems, Foster City, CA) were used to measure the mRNA levels of Wnt, β-catenin, and the housekeeping gene through RT-qPCR. The following primers were used:

GAPDH, (forward: 5′-TGCTGGTGCTGAGTATGTC GTG-3′ and reverse: 5′-CGGAGATGATGACCCTTTTGG- 3′, product size, 101 bp); Wnt, (forward: 5′- CCGTGACCTCTCTGTGTATC-3′ and reverse: 5′- TTCACCTCGGAAACACTTCA -3′, product size, 122 bp.); β-catenin, (forward: 5′-CAAGATGATGGTGTGCCAAG-3′ and reverse: 5′-CCATCTCAGCTTCCTGATGT-3′, product size, 140 bp).

According to the following protocol, each real-time PCR reaction was performed in a final volume of 20 μl of a mixture containing 2 μl of template cDNA, 1 μmol/L of each gene-specific primer, 6 μl of RNase-free distilled water, and 10 μl of SYBR Green Master Mix. Taq DNA polymerase was activated by pre-incubation at 95 °C for 15 min and 40 three-step cycles (32). Wnt and β-catenin gene expression were normalized against GAPDH expression. The relative quantity of gene expression was assessed using the comparative threshold cycle method (Ct) with arithmetic formulas (∆∆Ct^-2^).

***Statistical analysis***

Results were analyzed using GraphPad Prism 8 and expressed as mean ± standard error of the mean (SEM). Statistical evaluation was performed by one-way analysis of variance (ANOVA), and *P*<0.05 was considered significant. Also, Tukey’s test was used for pairwise comparisons. 

## Results

***ECG parameter analysis***

**Figure 1:**
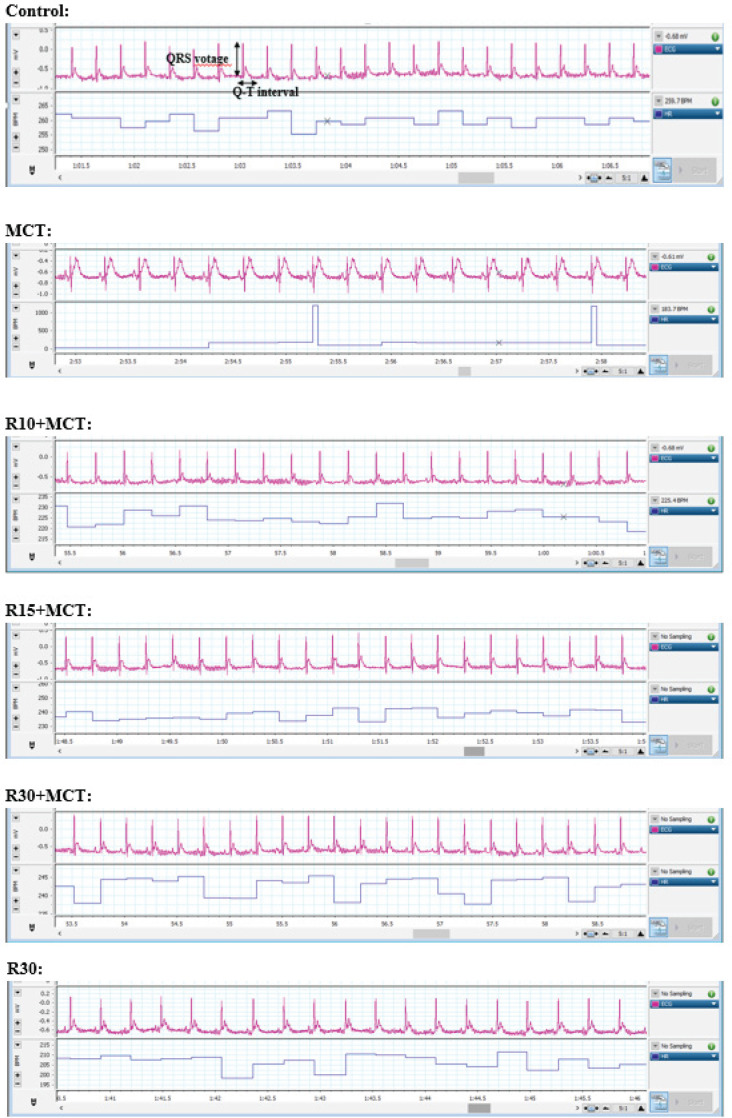
Effect of RA on the normal recording of lead II ECG in different groups of animal (n = 8): Control, MCT, R10+MCT, R15+ MCT, R30+ MCT, and R30

RA: Rosmarinic acid; ECG: Electrocardiography; MCT: Monocrotaline

The normal recording of lead II ECG is shown in Figure 1; compared to the control group, a drastic decrease in QRS complex voltage and an increase in QTc interval in the MCT group were quite clear. RA had an ameliorative effect on the above factors against the complications caused by the RVH model.

**Figure 2:**
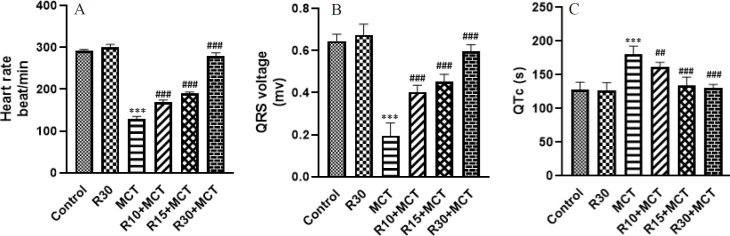
Effect of RA on electrocardiogram parameters in different groups of annimal

The HR in the MCT group was significantly lower than in the control group (*P*<0.001), as shown in Figure 2A. The animals in the R10+MCT, R15+MCT, and R30+MCT groups all had significantly higher heart rates than the animals in the MCT group (*P*<0.001).

When compared to the control group, the rats in the MCT group displayed a significantly lower voltage of the QRS complex (*P*<0.001). QRS complex voltage was significantly increased in the monocrotaline groups receiving RA (*P*<0.001) (Figure 2B).

As demonstrated in Figure 2C, when compared to the control group, the MCT group’s QTc interval significantly increased (*P*<0.001). RA was able to significantly reduce the QTc interval in the R10+MCT, R15+MCT, and R30+MCT groups (*P*<0.01, *P*<0.001, and* P*<0.001, respectively).

***Assay of hemodynamic functions***

**Figure 3:**
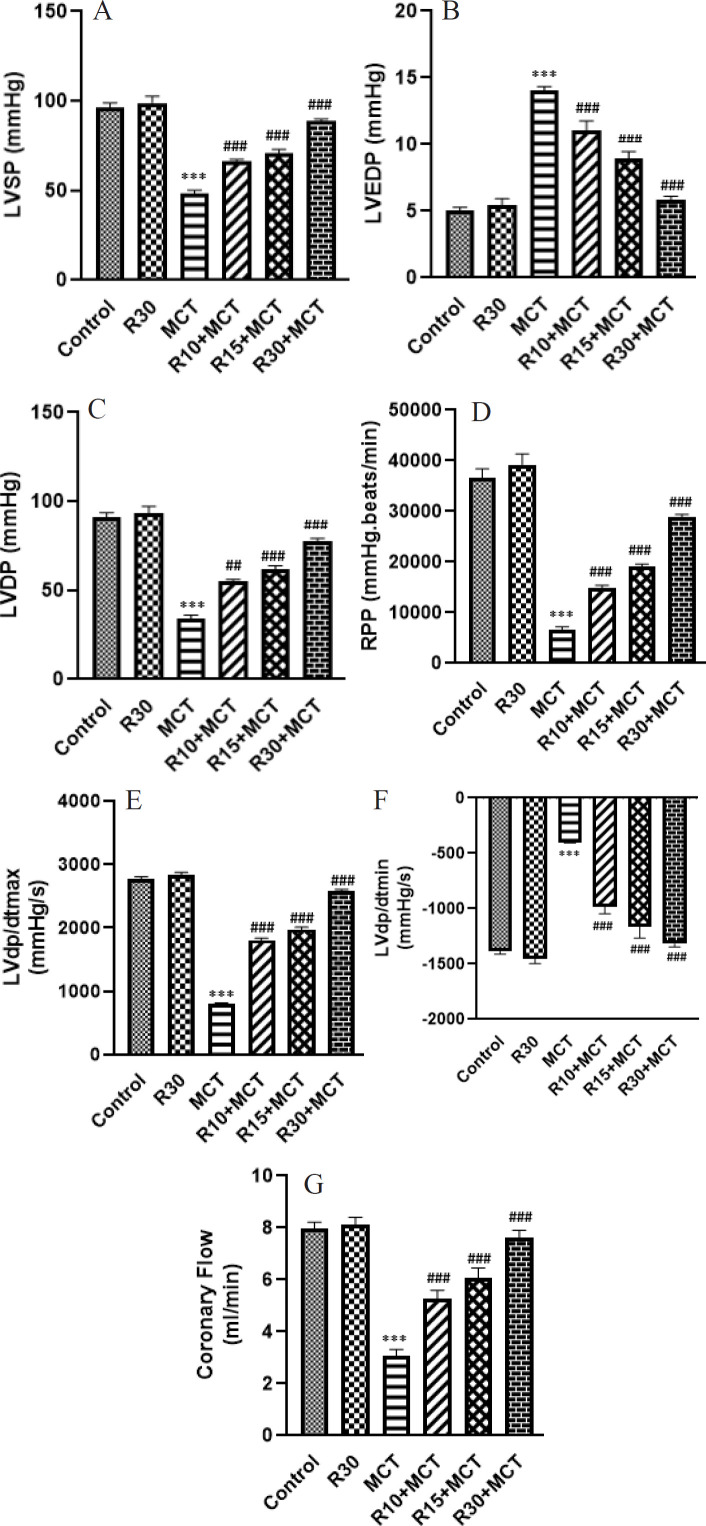
Effect of RA on A: LVSP, B: LVEDP, C: LVDP, D: RPP, E: LVdp/dtmax, F: LVdp/dtmin, and G: Coronary flow in different groups of animals

As demonstrated in Figure 3A, LVSP of the MCT group was significantly lower than that of the control group (*P*<0.001). Compared with the MCT group, LVSP was significantly higher in the rat R10+ MCT, R15+ MCT, and R30+ MCT groups (*P*<0.001).

As indicated in Figure 3B, MCT injection significantly raised LVEDP in the MCT group (*P*<0.001). RA significantly reduced LVEDP in R10+ MCT, R15+ MCT, and R30+ MCT groups (*P*<0.001).

A significant decrease in LVDP was observed in the MCT group compared with the control group (*P*<0.001). When compared to the MCT group, the LVDP in the rats of the R10+MCT, R15+MCT, and R30+MCT groups significantly (*P*<0.001) increased (Figure 3C).

As indicated in Figure 3D, there was a significant decrease in RPP (Rate pressure product), which depends on LVDP and HR, in the MCT group compared with the control group (*P*<0.001). When compared to the MCT group, RA was able to significantly raise the RPP in the rats from the R10+MCT, R15+MCT, and R30+MCT groups (*P*<0.001).

As illustrated in Figures 3E and F, myocardial contractility, represented by the maximum derivative of left ventricular pressure (LVdp/dtmax) and the minimum derivative of left ventricular pressure (LVdp/dtmin), decreased in the MCT group compared to the control group (*P*<0.001). RA improved the myocardial contractility significantly (*P*<0.001) in R10+MCT, R15+ MCT, and R30+ MCT groups.

As shown in Figure 3G, there was a significant decrease in coronary blood flow in the MCT group compared to the control group (*P*<0.001). In comparison to the MCT group, the groups receiving RA showed a significant increase in coronary blood flow (*P*<0.001).

***Anti-oxidant enzymes and MDA***

**Figure 4:**
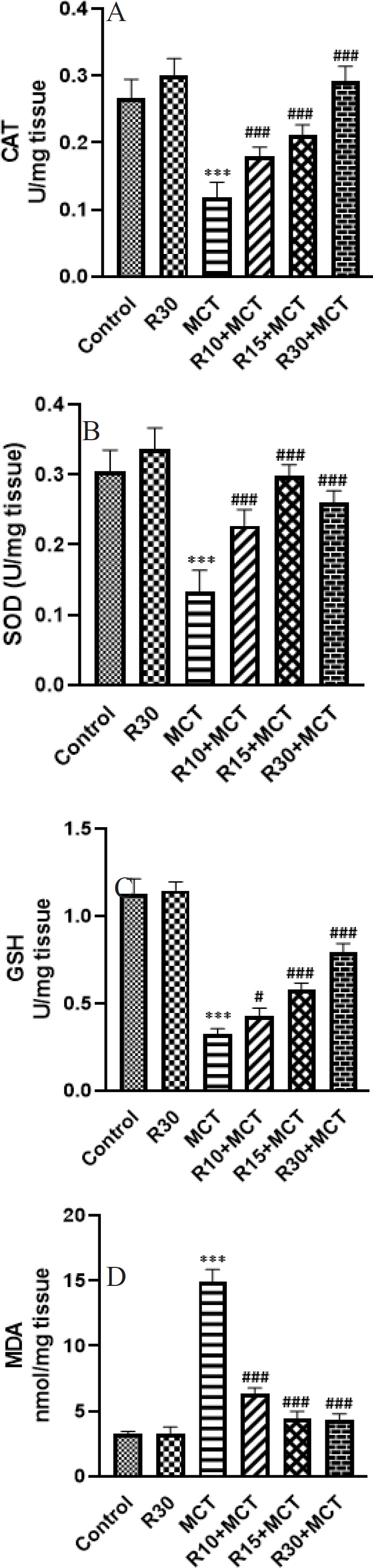
Effect of RA on A: CAT, B: SOD, C: GSH, and D: MDA. Results are expressed as mean ± SEM (n = 8) in different groups of animals

Compared to the control group, the activity of CAT in the heart tissue (left ventricle) of the MCT group significantly decreased (*P*<0.001). RA was able to significantly (*P*<0.001) improve this enzyme in the groups receiving RA (Figure 4A).

Comparing the MCT group to the control group, SOD activity in the heart tissue (left ventricle) was significantly reduced (*P*<0.001). In the groups that received RA, SOD activity increased significantly (*P*<0.001) (Figure 4B).

The GSH level in heart tissue (left ventricle) was significantly decreased in the MCT group (*P*<0.001). The rats in the R10+MCT, R15+MCT, and R30+MCT groups showed a significant increase in GSH levels compared to the MCT group (*P*<0.05 and *P*<0.001, respectively) (Figure 4C).

As indicated in Figure 4D, the MDA level in heart tissue (left ventricle) was significantly increased in the MCT group (*P*<0.001). RA caused a significant decrease in MDA levels in R10+ MCT, R15+ MCT, and R30+ MCT groups (*P*<0.001).

***Evaluation of RVHI measurement***

**Figure 5:**
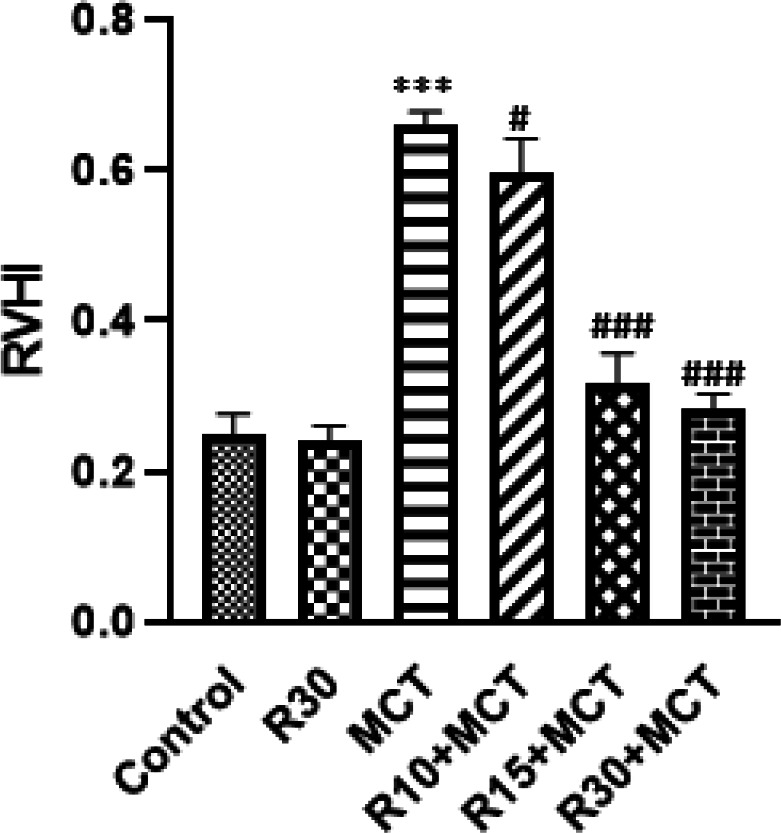
Effect of RA on RVHI in different groups of animals

Fulton’s index, a measure of right ventricular hypertrophy, significantly (*P*<0.001) increased in the MCT group. Compared with the MCT group, co-treatment with 10, 15, and 30 mg/kg RA resulted in significantly lower cardiac hypertrophy indices (*P*<0.05, *P*<0.001, and *P*<0.001, respectively) (Figure 5).

***Histology of heart tissue***

**Figure 6:**
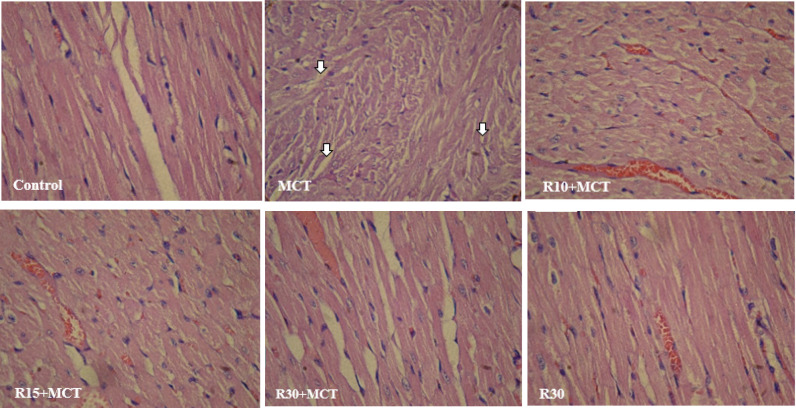
Effect of RA on histology of the heart tissue in different groups of animals (using H&E staining)

To investigate pathological changes in the rat myocardium in each treatment group, H&E staining was done. The structure and morphology of the cells and their nuclei were normal in the control group. On the contrary, in the MCT group, the intercellular substance expanded, and the myofibrils showed a disrupted and disordered arrangement. Co-treatment with RA significantly improved myocardial morphology in RA-receiving groups, especially in the R30 + MCT group. Myocardial fibers were connected and arranged more regularly, and the structure and morphology of the cells and their nuclei had a normal appearance. So, co-treatment with RA reduced these histopathological changes compared with the MCT group (Figure 6).

***Wnt/ β-catenin gene expression***

**Figure 7:**
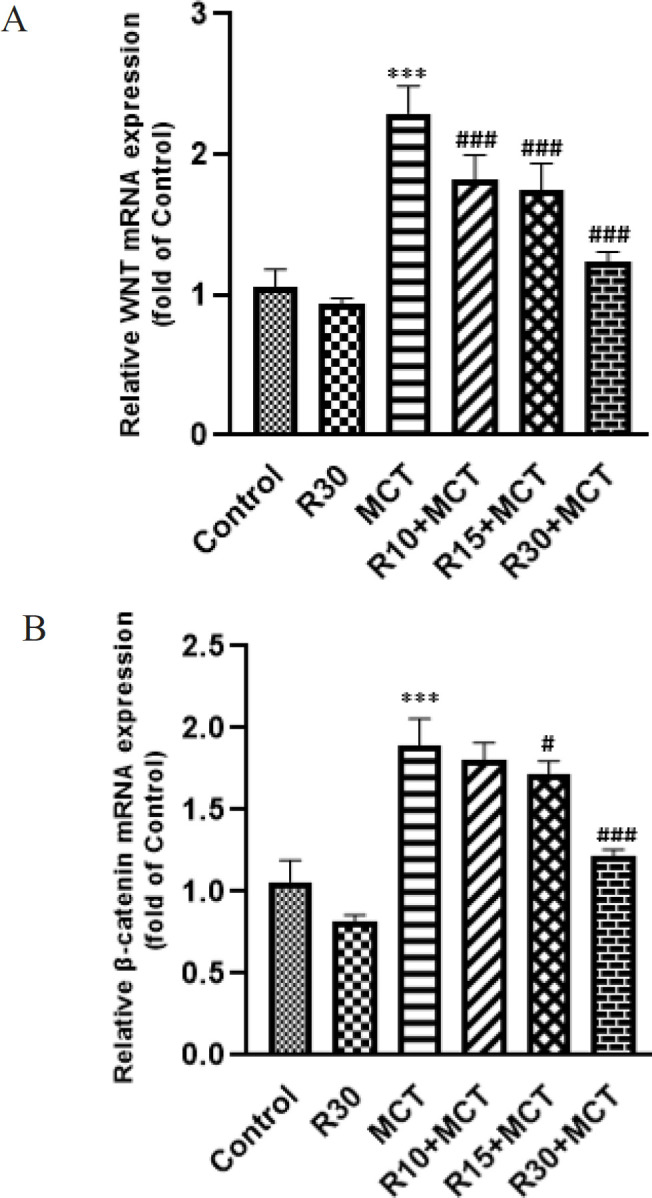
Effect of RA on Wnt/ β-catenin mRNA expression in different groups of animals

Using RT-qPCR, the levels of Wnt and β-catenin gene expression in the different groups were evaluated. As indicated in Figure 7A, the MCT group’s Wnt gene expression was significantly increased (*P*<0.001). RA was able to significantly decrease the expression of the Wnt gene in the RA-receiving groups (*P*<0.001).

Compared to the control group, there was a significant increase in the β-catenin gene expression in the MCT group (*P*<0.001). In the R10+MCT group, gene expression of β-catenin was slightly down-regulated compared with the MCT group. However, this change was not significant (*P*>0.05), whereas, in the R15+MCT and R30+MCT groups, gene expression was significantly decreased (*P*<0.05 and *P*<0.001, respectively) (Figure 7B).

A: Heart Rate, B: Voltage of QRS, and C: QTc interval. Results are expressed as mean ± SEM (n = 8). One-way ANOVA was used followed by Tukey’s test. ***P<0.001 vs Control group. ### *P*<0.001 and ## *P*<0.01 vs MCT group.

RA: Rosmarinic acid; MCT: Monocrotaline; QTc: Q-T corrected

Results are expressed as mean ± SEM (n = 8). One-way ANOVA was used followed by Tukey’s test. ***P<0.001 vs Control group, ### *P*<0.001 and ## *P*<0.01vs MCT group RA: Rosmarinic acid; LVSP: Left ventricular systolic pressure; LVDP: Left ventricular developed pressure; RPP: Rate pressure product; LVdp/dtmax: Left ventricular pressure; MCT: Monocrotaline

One-way ANOVA was used followed by Tukey’s test. ***P<0.001 vs Control group, ### *P*<0.001 and # *P*<0.05 vs MCT group.

RA: Rosmarinic acid; CAT: Catalase; SOD: Superoxide dismutase; GSH: Glutathione; MDA: Malondialdehyde; MCT: Monocrotaline

Results are expressed as mean ± SEM (n = 8). One-way ANOVA was used followed by Tukey’s test. ***P<0.001 vs Control group, ### *P*<0.001 and # *P*<0.05 vs MCT group.

RA: Rosmarinic acid; RVHI: Right ventricular hypertrophy index; MCT: Monocrotaline

In the MCT group, the intercellular substance was expanded, as indicated by arrows. Also, the myofibrils showed a disrupted and disordered arrangement, which is evident in the figure.

RA: Rosmarinic acid; MCT: Monocrotaline

Results are expressed as mean ± SEM (n = 8). One-way ANOVA was used followed by Tukey’s test. ***P<0.001 vs Control group, ### *P*<0.001 and # *P*<0.05 vs MCT group

## Discussion

This study aimed to determine whether co-treatment with RA could prevent left ventricular dysfunction in an experimental rat model of monocrotaline-induced RVH.

Our results demonstrated that administration of MCT caused an increase in RVHI, the QTc intervals, lipid peroxidation, and Wnt/β-catenin gene expression but decreased QRS complex voltage, heart rate, and anti-oxidant enzyme activities, and also caused significant changes in the hemodynamics, that administration of RA ameliorated all of the above-mentioned RVH-induced complications. 

Some toxicants, such as MCT, doxorubicin, and acetaldehyde, may induce cardiac hypertrophy by increasing oxidants, circulating catecholamines concentrations, and hemodynamic load, or by inducing hypoxia (33).

The previous studies confirmed that the liver is where MCT is metabolized and transformed into its active form. These active products damage the pulmonary vessels and increase vascular resistance, which causes pulmonary arterial hypertension and RVH (34).

Therefore, in the present study, we used an MCT-induced RVH model to evaluate the therapeutic effects of RA.

When RV is overstressed by pulmonary hypertension or RV outflow tract obstruction, progressive contractile dilation follows an initial adaptive response that involves RVH (35). It is well known that constant increases in pressure and size of RV lead to loss of contractile forces for blood circulation. Furthermore, given that persistent uncorrected RV failure often causes left ventricular dysfunction, leading to deleterious losses in cardiac output and ultimately heart failure, many therapeutic approaches to curing RVH are required (36). Therefore, many researchers are working to find treatments for RV dysfunction, and several animal models of RV dysfunction, including RVH, have been used. 

MCT causes destruction, and apoptosis of endothelial cells disrupts endothelial nitric oxide synthesis and nitric oxide signaling, and also induces oxidative stress responses (37). The outcomes of this study also demonstrated that injection of MCT causes cardiovascular oxidative stress damage by increasing MDA and reducing the efficiency of the anti-oxidant defense system. Oxidative stress is among the important elements in the pathogenesis of cardiovascular diseases. Therefore, the increase in MDA as a response to decreased anti-oxidant defense response may contribute to the development of cardiovascular complications, which agrees with the results obtained in our study. 

Our findings are consistent with earlier research showing adverse effects on hemodynamic functions in MCT-treated rats (38).

Since the oxidative stress and heart failure caused by monocrotaline-induced RVH affect the whole heart (39), also due to the role of the left ventricle in systemic blood circulation, in the current experiment, the effects of RVH on the hemodynamic function of the left ventricle including LVSP, LVEDP, LVDP, RPP, LVdp/dtmin, LVdp/dtmax, and coronary flow in isolated rat hearts were investigated. The effects of RVH induced by MCT on the isolated rat heart were associated with suppression of contractile function as indicated by decreased LVDP, an impaired inotropic force of contraction, myocardial relaxation, and increased LVEDP (40). Increased LVEDP resulted in low perfusion in the subendocardial region of the heart, which undergoes the most severe compression during systole, ultimately aggravating ischemic injury (41). In this study, RA significantly protected hemodynamic parameters and preserved ventricular function at the three doses tested. This is shown by improvements in LVdp/dtmax and LVdp/dtmin, which indirectly suggests that an increase in max/min LVdp/dt and ventricular filling leads to an increase in cardiac output. Due to improved cardiac contractility, LVEDP decreases and leads to increased subendocardial blood flow (42, 43). In this study, the groups that received RA showed a significant improvement in cardiac function, which may be related to its anti-oxidant properties.

Oxidative stress and inflammation are the two main mechanisms responsible for vascular remodeling (44). Numerous studies on the oxidative stress induced by MCT injection show a decrease in glutathione (GSH) and an increase in malondialdehyde (MDA) (45). In this study, the measurement of SOD, CAT, and GSH enzymes in the heart tissue of MCT rats showed a decrease.

Past studies have shown that the most widely used tool for detecting the presence of RVH is electrocardiography (ECG) (46). We discovered in the current study that MCT administration in the MCT group decreased QRS voltage and QTc interval prolongation. We found that RA co-treatment was successful in increasing QRS voltage and reducing QTc interval prolongation, indicating the protective effect of RA on the cardiovascular complications of RVH.

In VSMCs, in an oxidative environment, phenotypic changes occur that can lead to vascular inflammation and calcification, which can lead to vascular dysfunction (47). As previously mentioned, vascular calcification is a threatening complication of cardiovascular diseases that affects tissues such as arteries, heart valves, and the heart muscle (9). Previous research has demonstrated that the Wnt/β-catenin signaling pathway is involved in the osteoblastic differentiation of mesenchymal stem cells. Since osteoblasts and vascular smooth muscle cells (VSMCs) share the same mesenchymal origin, the osteogenic transformation of VSMCs resembles bone formation. Additionally, there is enough evidence to demonstrate the crucial part that Wnt/β-catenin signaling plays in vascular calcification (13). For this reason, we investigated the gene expression of Wnt/β-catenin in the heart. The results obtained in our study showed that MCT injection increased the expression of the Wnt/β-catenin gene in the MCT group and co-treatment with RA decreased its expression.

## Conclusion

Generally, the results of this study showed that RVH induced by MCT causes oxidative stress and complications on the left ventricle in laboratory animals. We also demonstrated that co-treatment with RA, a natural anti-oxidant, prevented cardiovascular complications induced by RVH and protected cardiac structural changes. In light of these findings, it can be said that co-treatment with RA prevents cardiac dysfunction caused by RVH by increasing anti-oxidant activity and decreasing the expression of Wnt and β-catenin genes.

## Authors’ Contributions

M D and N A were responsible for the design of this project. M D and M R designed the experiments. N A and M R collected and processed the data. M B and SA M analyzed and interpreted the results. N A wrote the manuscript. The authors declare that all data were generated in-house and that no paper mill was used.

## Conflicts of Interest

All authors declare no conflicts of interest.
